# 

**Published:** 1865-10

**Authors:** 


### Editorial.

### THE OHIO COLLEGE OF DENTAL SURGERY.

The regular course of instruction in this institution commences on the 1st Monday in November, and will continue to the last of February. The Infirmary and Laboratory will be open under the supervision of their respective teachers, for the reception of students, on and after the 16th of this month (October).

There will also be in addition, one lecture daily, till the commencement of the regular session.

It is very desirable that all students who can, should avail themselves of this preliminary course, though it seems but short, they will find it greatly advantageous; they will, during that time, become somewhat familiar with the method of procedure, and have things so arranged that when the regular session begins, they can take hold, with energy and undivided attention, of the work that is before them. There will be every facility for a most thorough course. Very extensive clinical advantages will be available.

The dental profession seem to manifest a far greater interest in the college now than ever before, and in this we see indicated, an extended sphere of usefulness, for the Ohio College of Dental Surgery in the future.

### The Dental Register

OF THE WEST.

*Issued Monthly, at $3 a Year in Advance.*

DEVOTED TO THE INTERESTS OF THE DENTAL PROFESSION.

EDITED BY J. TAFT

The volume commences in January and closes in December.

The Register being issued monthly is always fresh, therefore indispensable to the practitioner who desires to keep posted up with the new inventions and improvements which are daily developed. Neither expense nor effort will be spared to give value and variety to its contents. Articles will be illustrated with well executed engravings whenever they will add to the interest or assist in explanation.

Its extensive circulation and frequency of issue makes it one of the BEST DENTAL ADVERTISING MEDIUMS in the United States.

RATES OF ADVERTISING.

One page, 1 year, $50. Half page, 1 year, $30. Quarter page, or card, 1 year $20.

All advertising bills payable in advance, or at furthest after the issue of the first number ontaining the advertisement. All transient advertisements to be paid for in advance.

☞ CONTRIBUTIONS SOLICITED.

Address, J. TAFT, or "DENTAL REGISTER," Cincinnati, Ohio.

Business Communications to

**J. TAFT, Publisher,**

No. 172 Race Street.

### ROBERTS' OS-ARTIFICIAL.

A substitute for AMALGAM in filling badly decayed teeth; and used for reacting Pivot Teeth in badly decayed roots, also for filling over SENSITIVE DENTINE to destroy sensibility, and as a non-conductor of heat, and for many other dental purposes.

For sale by all dealers in Dental Materials, and by the undersigned.

One-fourth ounce packages with directions sent by mail free of postage on receipt of $1.

**C. H. ROBERTS, M. D.**

**POUGHKEEPSIE, N. Y.**

